# A novel approach to assessing disparity in representativeness of clinical trial participants within a large midwestern healthcare system

**DOI:** 10.1016/j.conctc.2024.101274

**Published:** 2024-02-14

**Authors:** Anne Rivelli, Cheryl Lefaiver, Maureen Shields, Osondi Ozoani-Lohrer, Andy Marek, Jana Hirschtick, Veronica Fitzpatrick

**Affiliations:** a Advocate Aurora Research Institute, Milwaukee, WI, USA; b Center for Child and Family Research, Milwaukee, WI, USA; c Advocate Health, Milwaukee, WI, USA

**Keywords:** Clinical trial, Disparity, Representativeness, Clinical trial participation

## Abstract

**Background:**

Representativeness in clinical trials (CT) serves as a metric of access to healthcare and reflects differences that may determine differential efficacy of medical interventions; thus, quantifying representativeness in CT participation is critical.

**Methods:**

This retrospective, descriptive study utilized patient demographic data extracted from the largest Midwestern non-profit healthcare system. Using data between January 1, 2019 and December 31, 2021, a CT Participant Sample of 4,537 system patients who were active CT participants was compared to a CT Patient Population of 195,726 system patients receiving care by the PI of active CTs, which represented the target population. Chi-square goodness-of-fit tests were used to test differences in distributions of demographic variables between groups, indicating disparity in CT participation. Two metrics adapted from literature - participation incidence disparity (PID) and participation incidence ratio (PIR) - were calculated to quantify absolute and relative disparity in representativeness proportions, respectively. Descriptive approaches to assessing representativeness are also provided.

**Results:**

Results showed significant differences by race/ethnicity (χ2 = 50.64; p < 0.0001), age categories (χ2 = 56.64; p < 0.0001), and insurance (χ2 = 41.29; p < 0.0001). PID and PIR metrics revealed reduced CT participation among non-White racial/ethnic groups and increased CT participation among White Non-Hispanic patients. Further, CT participants ≥80 or Worker's Compensation were underrepresented while those with Self-Pay insurance were overrepresented as CT participants.

**Conclusions:**

Despite progress, continued efforts to not only enroll participants into CTs that are representative of the healthcare system and region, but also to better assess representativeness quantitatively are still needed.

## Highlights

• Assessing clinical trial (CT) representativeness is critical.
• This study offers a comprehensive framework to assess CT representativeness.
• Data offer descriptive and quantitative approaches to assess representativeness.
• Findings support differences in participation by race/ethnicity, age and insurance.
• Findings from this large healthcare system inform progress at other institutions.

## 1 Background

Clinical trials (CT) offer the most robust evidence of efficacy and safety of novel medical interventions [1]. Evidence gained from CT is used to establish (or re-establish) effective approaches to medical care and, further, to determine allocation of resources and guide funding priorities. Thus, for equitable medical care, it is critical that the evidence gained from CTs represent all groups of people eligible and needing such care [2,3]. Disparity describes differences in health outcomes or their determinants between groups, as defined by demographic and other characteristics [4]. Disparity in representativeness can lead to erroneous and/or disparate outcomes between groups, and efforts to reduce disparities promote progress toward health equity [2,5].

Representativeness in CTs serves as a metric of societal access to healthcare while simultaneously allowing for measurement of biologic differences like age and sex and social factors, including race/ethnicity, that may determine differential efficacy of medical interventions [6]. Unfortunately, trial populations frequently do not represent the populations they are intended to emulate [6,7]. Representativeness is a critical concept in healthcare research, as it is a measure of external validity, or how well the outcome(s) of a research study can be generalized to the greater population being studied. A recent literature review on the representativeness of randomized CT (RCT) samples, assessed by comparing the characteristics of a RCT sample with those of everyday clinical practice patients, concluded that RCT samples, overall, were not representative of patients encountered in clinical practice [7]. While two studies found no differences in demographics between RCT participants and their patient populations [8,9], most studies showed demographic differences reflecting RCT participants who were more often older [[10], [11], [12], [13], [14], [15], [16], [17], [18], [19], [20], [21], [22], [23]], female [[10], [11], [12], [13], [14], [15],^17^,^24^], male [25], or Caucasian [26]. As evidenced by this literature review, there has been a long history of non-representativeness of CT samples on baseline demographic characteristics that has only begun to make small shifts in the past 30 years [7].

Attaining appropriate representation in CTs requires intentional effort due to the history of disparity within healthcare systems. Major organizations and funding institutions, like the NIH, have implemented initiatives designed to foster the inclusion of underrepresented groups in NIH-supported clinical research trials [27]. Similarly, the FDA implemented reporting requirements and issued a recommendation for sponsors of clinical trials to increase enrollment of underrepresented populations [28]. Thus, it is critical to monitor progress toward CT participant representativeness in healthcare institutions and systems.

Current approaches to assessing representativeness sampling vary, but the goal is the same: to calculate representation metrics for all subgroups and use visualization and statistical methods that enable effective identification of significantly underrepresented subgroups with respect to the target population [29]. While there is no agreed upon assessment method or quantitative metric(s) to determine representation, the two commonly used approaches include comparing important outcomes or characteristics of the CT sample with those of everyday clinical practice patients and assessing what proportion of a real-world population would satisfy the criteria for CT inclusion [7]. In both approaches, the identification of a target population based on appropriate real-world data sources, such as electronic health record or a regionally- or nationally-representative population sample with which to compare a sample, is critical [29,30]. Further, metrics, such as the absolute or relative difference between groups, act as important indicators to quantify representativeness [6,29]. Additionally, as with all research, the target population must be clearly defined, with clarity on assumptions being made and known limitations in what the population represents and how results are being applied [5,30].

The purpose of this study is to comprehensively assess the demographic representativeness of the largest midwestern healthcare system's sample of active clinical trial participants between January 1, 2019 and December 31, 2021 as compared to a target population. The target population includes all patients treated by the providers serving as Principal Investigators (PIs) of all active clinical trials in the same time period. This study leveraged a clinical trial management system data (CTMS) as well as electronic medical record data (EMR) from one of the largest healthcare systems in the U.S. to capture data needed to comprehensively assess representativeness. Representativeness in such a large healthcare system can inform strides being made toward representativeness in other healthcare institutions and systems. Further, this study provides a novel framework for assessing representativeness, reflecting standard methods while also incorporating additional data and metrics with which to quantify disparities.

## 2 Materials and methods

This retrospective, descriptive study utilizes patient demographic data from two groups extracted from CTMS and EMR within the largest Midwestern non-profit healthcare system. The first dataset includes data of a sample of 4,537 healthcare system patients who provided informed consent to participate in at least one active CT within the system between January 1, 2019 and December 31, 2021 (“CT Participant Sample”). The second dataset includes data of the 195,726 healthcare system patients who were receiving care by at least one PI of an active CT within the system between January 1, 2019 and December 31, 2021 (“CT Patient Population”). The CT Patient Population was extracted to represent the target population, or the comparative greater population from which the CT Participant Sample was drawn. As such, it includes all patients presumably eligible to participate in an active CT. Additionally, publicly-available data reflecting demographic proportions across the United States (U.S.) Midwestern region population (“Midwest Region”) are provided to be viewed descriptively as a complementary approach to assessing representativeness. It should be noted that the Midwest Region represents estimates of all individuals in the Midwest region, comprising those receiving and not receiving healthcare. This comparative population was chosen because the healthcare system is based in the Midwest region and primarily serves individuals who live regionally.

### 2.1 Data

Demographic variables were collected from CTMS and EMR on the CT Participant Sample and the CT Patient Population, including: Sex (categorical), Male or Female; Race/ethnicity (categorical), White Non-Hispanic or Latino (NH), Black NH, Asian NH, American Indian or Alaskan Native NH, Native Hawaiian and Other Pacific Islander NH, and Hispanic or Latino; Insurance type (categorical), Private, Medicare, Medicaid, Self-pay, Worker's Comp; and date of birth to calculate age (continuous) as of January 1, 2019 for both groups. Demographic variables were captured from CTMS at clinical trial enrollment for the CT Participant Sample or from EMR at first encounter in the timeframe (January 1, 2019–December 31, 2021) for the CT Patient Population. Finally, demographic proportions of corresponding variables across all individuals in the U.S. Midwest Region were gathered from two sources [31,32]. While most patients in the healthcare system reside in Illinois and Wisconsin, the Midwest Population proportions reflect 12 states, specifically: Illinois, Wisconsin, Indiana, Michigan, Ohio, Missouri, Minnesota, Iowa, Kansas, Nebraska, South Dakota and North Dakota.

### 2.2 Statistical methods

Data management and analysis of the sample were conducted with SAS statistical software (Version 9.4; SAS Institute, Cary, NC). All analyses of the sample data were performed by research personnel employed by the health system. Proportions were calculated across all demographic variable levels in both datasets. Additionally, 95% confidence intervals were calculated for the CT Participant Sample proportions.

A Chi-square goodness-of-fit test was used to test whether the frequency distribution of demographic variables in the CT Participant Sample was significantly different from the CT Patient Population, as this analysis offers a standard approach to compare an observed proportion to a population proportion [6,33]. In this case, Chi-square goodness-of-fit tests were used to determine how well subgroups of the CT Participant sample generalized to the target population comprised in the CT Patient Population. We tested the null hypotheses that the observed proportions in the CT Participant Sample were equal to the known proportions drawn from the demographic makeup of the CT Patient Population. Further exploration was pursued to identify levels within each variable that were contributing most meaningfully to Chi-square parameter itself (“ChiSqContrib”) and proportion (“ChiSqPropor”) of the parameter. A two-sided alpha of p < 0.05 was considered statistically significant.

To offer additional avenues to assess disparity in representativeness, we calculated two metrics adapted from published literature that reflect absolute and relative differences in observed and expected group proportions to quantify representativeness [6,29]. First, participation incidence disparity (PID) is calculated as the absolute difference between the proportion of patients of a demographic level variable among the CT Participant Sample and the proportion of patients of that same demographic level variable in the CT Patient Population. Second, participation incidence ratio (PIR) is calculated as the ratio of proportions of patients in a demographic category within the CT Participant Sample vs. the CT Patient Population. PIR mirrors the disparate impact measure, which has been proposed as an intuitive quantity similar to the four-fifths, or 80 Percent rule, suggested by the United States Equal Employment Opportunity Commission guideline of 80% as the parameter to decide if the result is unfair [29,34]. The disparate impact measure reduces to the equivalent of the ratio of observed participation proportion of a subgroup to the proportion of another subgroup. When applied here, the demographic distribution of the CT Participant Sample must be at least 80% of that of the CT Patient Population to be deemed “representative”, “fair”, and without disparate impact. In this context, disparate impact reflects reduced participation (as defined by <80%) of specific demographic groups within the CT Participant Sample compared to those demographic groups within the CT Patient Population. Analogously, disparate impact of >120% reflects increased participation of specific groups within the CT Participant Sample as compared to the CT Patient Population.

## 3 Results

Table 1 describes proportions across demographic variables among the CT Participant Sample (N = 4,537), CT Patient Population (N = 195,726) and Midwest Region in 2021 (N = 68,841,440), respectively.

**Table 1:** Demographic distributions of CT participant sample, CT patient population, and Midwest region.^a^

	*CT Participant Sample (January 1, 2019-December 31, 2021)* N = 4,537	*CT Patient Population (January 1, 2019-December 31, 2021)* N = 195,726	*Midwest Region Population (2021)*^b^ N = 68,841,440
*Sex*			
Female	2,566 (56.59%)	110,594 (56.50%)	34,420,720 (50.00%)
Male	1,968 (43.41%)	85,132 (43.50%)	34,420,720 (50.00%)
*Race/Ethnicity*			
Hispanic or Latino	257 (5.85%)	15,090 (7.84%)	5,507,315 (8.00%)
AI/AN NH	17 (0.38%)	1,046 (0.54%)	0 (0.00%)
Asian NH	90 (2.03%)	5,412 (2.81%)	2,065,243 (3.00%)
Black NH	521 (11.73%)	24,747 (12.85%)	6,884,144 (10.00%)
NH/PI	4 (0.09%)	412 (0.21%)	0 (0.00%)
White NH	3,553 (79.99%)	145,857 (75.74%)	50,254,251 (73.00%)
*Age Category*			
18.0 < 30.0	286 (7.12%)	13,427 (6.86%)	8,949,387 (13.00%)
30.0 < 40.0	385 (9.58%)	16,311 (8.33%)	8,949,387 (13.00%)
40.0 < 50.0	396 (9.86%)	21,433 (10.95%)	8,260,972 (12.00%)
50.0 < 60.0	694 (17.28%)	37,816 (19.32%)	8,949,387 (13.00%)
60.0 < 70.0	1,054 (26.24%)	49,345 (25.21%)	8,949,387 (13.00%)
70.0 < 80.0	882 (21.96%)	37,613 (19.22%)	5,507,315 (8.00%)
≥80.0	320 (7.97%)	19,781 (10.11%)	2,753,657 (4.00%)
*Insurance*			
Medicaid	371 (8.30%)	19,179 (9.86%)	12,033,483 (17.48%)
Medicare	2,311 (51.71%)	104,272 (53.63%)	10,505,203 (15.26%)
Private	1,584 (35.44%)	64,246 (33.04%)	36,582,341 (53.14%)
Self-Pay	192 (4.30%)	6,134 (3.15%)	4,770,711 (6.93%)
Worker's Comp	11 (0.25%)	615 (0.32%)	–

a Demographic proportions are provided to be viewed descriptively as a complementary approach to assess representativeness.

b Midwest Region reflects 2021 population estimates from 12 states: North Dakota, South Dakota, Nebraska, Kansas, Iowa, Missouri, Minnesota, Wisconsin, Michigan, Ohio, Illinois, Indiana and reflect proportion rounded estimates documented by the 2021 Census Bureau [31] (sex, race/ethnicity, age) and 2021 Kaiser Family Foundation [32] (insurance). Cells reporting 0.00% reflect rounding.

Chi-square goodness-of-fit test results showed significant differences between the CT Participant Sample and the CT Patient Population by race/ethnicity, age categories, and insurance. Specifically, the Chi-square goodness-of-fit test showed that the racial/ethnic distribution among the CT Participant Sample statistically differed from the CT Patient Population (χ2 = 50.64; df = 4; p < 0.0001). Looking closer, disparity in the proportion of Hispanic or Latino individuals contributed most meaningfully to the Chi-square parameter (ChiSqContrib = 24.88), with 60.30% of the parameter proportion due to this level. The Chi-square goodness-of-fit test showed that the distribution of age categories among the CT Participant Sample statistically differed from the CT Patient Population (χ2 = 56.64; df = 6; p < 0.0001). Looking closer, disparity in the two oldest age category levels (70.0 < 80.0 and ≥80) contributed most meaningfully to the Chi-square parameter (ChiSqContrib = 15.65 and 18.26, respectively), with 27.60% and 32.20%, respectively, or 59.80% total of the parameter proportion due to these levels. Finally, the Chi-square goodness-of-fit test showed that the distribution of insurance levels among the CT Participant Sample statistically differed from the CT Patient Population (χ2 = 41.29; df = 4; p < 0.0001). Looking closer, differences in Self-Pay followed by Medicaid contributed most meaningfully to the Chi-square parameter (ChiSqContrib = 18.64 and 11.01, respectively), with 45.10% and 26.70% respectively, or 71.80% total of the parameter proportion due to these levels. No significant differences in sex distribution were found between the CT Participant Sample and the CT Patient Population (χ2 = 0.02; df = 1; p = 0.8977).

**Fig. 1:**
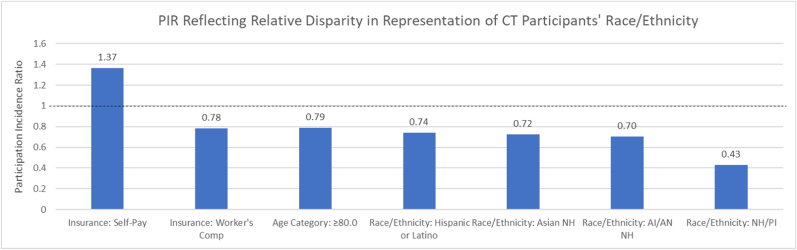
PIR reflecting relative disparity in representation of CT participants' race/ethnicity.

To quantify the representativeness of the CT Participant Sample, PIR and PID values reflect absolute and relative differences in proportions of patients of each demographic level among the CT Participant Sample vs. the CT Patient Population. Per the PIR calculations, using <80% and >120% to define disparate impact, there was relative disparity in representation across many racial/ethnic groups; specifically, Hispanic or Latino, Asian NH, AI/AN NH and NH/PI NH patients were underrepresented as CT participants (PIR = 0.74, 0.72, 0.70, and 0.43, respectively). Further, individuals in the CT Participant Sample with Worker's Compensation insurance and in the ≥80 age group were also underrepresented while those with Self-Pay insurance were overrepresented as compared to their counterparts in the CT Patient Population (PIR = 0.79, 0.78 and 1.37, respectively). See Table 2 and Fig. 1 for complete data and visualizations of levels indicating disparate impact, respectively.

**Table 2:** One-sample chi-square goodness-of-fit test results reflecting representativeness of CT participant sample to CT patient population.

	Sample% (95% CI)	Population%^a^	P-Value	ChiSqContrib	ChiSqPropor	PID^b^	PIR^c^
*Sex*							
Female	56.59 (55.15, 58.04)	56.50	0.8977	0.01	0.44	0.09	1.00
Male	43.41 (41.96, 44.85)	43.50		0.01	0.57	−0.09	1.00
*Race/Ethnicity*							
AI/AN NH	0.38 (0.20, 0.56)	0.54	<0.0001	2.11	0.05	−0.16	0.70
Asian NH	2.03 (1.61, 2.44)	2.81		10.08	0.24	−0.78	0.72
Black NH	11.74 (10.78, 12.68)	12.85		4.94	0.12	−1.11	0.91
Hispanic or Latino	5.79 (5.10, 6.47)	7.84		24.88	0.60	−2.05	0.74
White NH	80.06 (78.81, 81.16)	75.74		8.36	0.20	4.32	1.06
NH/PI NH^d^	0.09 (0.00, 0.18)	0.21	–	–	–	−0.12	0.43
*Age*							
18.0 < 30.0	7.12 (6.32, 7.91)	6.86	<0.0001	0.40	0.01	0.26	1.04
30.0 < 40.0	9.58 (8.67, 10.49)	8.33		7.59	0.13	1.25	1.15
39.0 < 50.0	9.86 (8.94, 10.78)	10.95		4.37	0.08	−1.09	0.90
50.0 < 60.0	17.28 (16.11, 18.45)	19.32		8.68	0.15	−2.04	0.89
60.0 < 70.0	26.24 (24.88, 27.60)	25.21		1.69	0.03	1.03	1.04
70.0 < 80.0	21.96 (20.68, 23.24)	19.22		15.65	0.28	2.74	1.14
≥80.0	7.97 (7.13, 8.80)	10.11		18.26	0.32	−2.14	0.79
*Insurance*							
Medicaid	8.30 (7.49, 9.11)	9.86	<0.0001	11.01	0.27	−1.56	0.84
Medicare	51.71 (50.25, 53.18)	53.63		3.07	0.07	−1.92	0.96
Private	35.44 (34.04, 36.85)	33.04		7.82	0.19	2.40	1.07
Self-Pay	4.30 (3.70, 4.89)	3.15		18.64	0.45	1.15	1.37
Worker's	0.25 (0.10, 0.39)	0.32		0.76	0.02	−0.07	0.78

a Reflects observed proportion in CT Patient Population.

b Participation Incidence Disparity (PID) is absolute proportion difference of CT Participant Sample and CT Patient Population across each demographic variable level.

c Participation Incidence Ratio (PIR) is the proportion of each demographic variable level in CT Participant Sample divided by that level in CT Patient Population.

d Native Hawaiian/Pacific Islander Non-Hispanic not included in Chi-square analysis due to cell count <5.

**Fig. 2:**
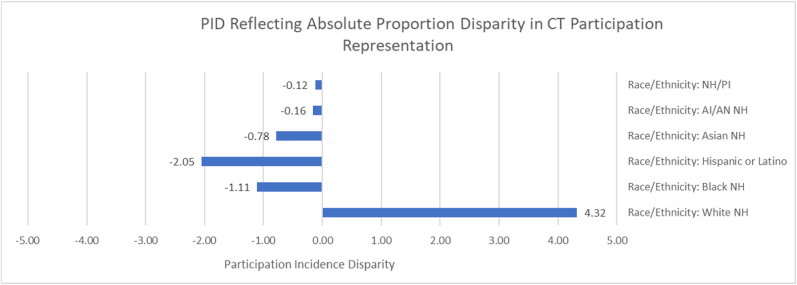
PID reflecting absolute proportion disparity in CT participation representation.

PID calculations among demographic categories of the CT Participant Sample reveal that the most widespread absolute proportion disparity is reflected among the racial/ethnic groups, specifically that, while the proportion of White NH individuals in the CT Participant Sample is 4.32 percentage points greater than the proportion in the CT Patient Population, the proportions of Hispanic or Latino, Black NH, Asian NH, AI/AN NH and NH/PI NH individuals in the CT Participant sample are 0.12%–2.05% percentage points lower than those in the CT Patient Population (PID = −2.05, −1.11, −0.78, −0.16, −0.12, respectively). Generally, White NH participants are overrepresented while all non-White groups are underrepresented in the CT Participant Sample. See Fig. 2 for a visual of levels reflecting relative disparity in representation.

## 4 Discussion

This study was performed using three data sources, including two EMR datasets from a large Midwestern U.S. health system including patients who participated in an active CT between January 1, 2019 and December 31, 2021 and one representing estimated proportions reflecting all individuals in the Midwest Region. The focus of this paper was on the comparison between the CT Participant Sample and the CT Patient Population, which represented the target population for representativeness. The goal of this study was to assess representativeness of CT participants in a large healthcare system to evaluate existing disparities in CT participants. The CT Participant Sample was compared statistically to the CT Patient Population and descriptively to the Midwest Region population to offer a comprehensive approach to assessing representativeness. This study was also intended to add to the literature the application of different metrics that can be used to assess representativeness that are not widely used in examining representation in sampling.

Regarding race/ethnicity representativeness, descriptively, proportions of all non-White groups in the CT Participant Sample – Hispanic or Latino and Non-Hispanic or Latino, American Indian/Alaskan Native, Asian, Black and Native Hawaiian/Other Pacific Islander – were lower than their counterparts in the CT Patient Population; conversely, there was a higher proportion of White NH individuals in the CT Participant Sample compared to the CT Patient Population. PID and PIR values also indicate disparities in race/ethnicity. While the PID values by race/ethnicity appear negligible at first glance, these values reflect actual patients from underrepresented groups who may have been denied an opportunity to receive treatment being offered in a CT. Translating percentages into numbers of patients, to match proportion of participants to the population proportions, CT should have included exactly 91 more Hispanic or Latino patients from the population, 49 more Black NH, 34 more Asian NH, 7 more AI/AN NH, 5 more NH/PI NH, and also enrolled 188 fewer White NH participants. Current literature reports similar amounts of under-enrollment of minorities with modest improvement over time [35,36].

Regarding insurance representativeness, greater proportions of privately-insured and self-pay individuals in the CT Participant Sample and lesser proportions of publicly-insured (Medicaid and Medicare) and Worker's Compensation individuals participated in CTs relative to those in the CT Patient Population. Interestingly, it is clear from the demographic distributions that, while privately-insured individuals are much more common in the Midwest Region, those participating and eligible for CTs within the healthcare system are much smaller proportions, suggesting fewer privately-insured individuals are seeking care for conditions utilizing CTs. This contrasts with Medicare patients, who are a smaller proportion of the Midwest Region as compared to the individuals in the CT Participant Sample and CT Patient Population, which is likely a function of the association between age and illness. Overall, the Midwest Region has smaller proportions of older patients relative to the CT Participant Sample and CT Patient Population, specifically individuals ages 50–80+, indicating more older individuals are seeking healthcare for conditions with available CTs and, further, participating in CTs.

Regarding age representativeness, in the CT Participant Sample, there were higher proportions of individuals ages <40 and ages 60–80 but lower proportions of individuals ages 40–60 and ≥ 80 relative to their counterparts in the CT Patient Population. Individuals in the CT Participant sample ages ≥80 showed disparate impact, or a PID <80%, indicating they were underrepresented as compared to proportion of patients ages ≥80 in the CT Patient Population. Interestingly, participants ages 70 < 80 were overrepresented compared to the patient population. This is likely explained by an increase in comorbidities and health complications with age that may exclude the oldest group of patients from eligibility and participation in CTs.

Finally, regarding sex representativeness, there are more females in the CT Participant Sample compared to males, which aligns with current literature [[8], [9], [10], [11], [12], [13], [14],^16^,^18^,^22^] and also closely reflects the distribution of patients seen by sex in the CT Patient Population.

Overall, statistical tests reflect disparity in representation across race/ethnicity, age, and insurance. Further exploration of Chi-square contributor proportions reveal that most disparity in representation is among individuals in the CT Participant Sample who identify as Hispanic or Latino, with a substantially smaller proportion of this demographic group participating in clinical trials relative to those being treated and presumably eligible, as represented by the CT Patient Population. It is possible that additional requirements for limited English proficient participants (e.g. translated documents, interpreter and witness for informed consent processes) impacted the large disparity for Hispanic or Latino and Asian individuals in the CT Participant Sample. Further, the perception of trials differs based on historic influences among groups. One group may view trials as an opportunity or gift, whereas another may have reservations about being “experimented upon” [6]. The literature suggests that minority populations are willing to participate in current and future CT when asked [[37], [38], [39]], however, investigators and research professionals may hesitate to offer CT to minorities because of perceptions and negative stereotypes associated with minorities [40]. Regardless, underrepresented groups face numerous barriers to CT participation, both external and internal.

### 4.1 Limitations

One of the challenges to assessing representativeness is identifying an “ideal” target population with which to compare a drawn sample. In healthcare, regional and national geographic populations do not necessarily represent individuals receiving healthcare or those eligible for CTs. For this reason, our analysis compared the CT Participant Sample to the CT Patient Population but did not compare the CT Participant Sample or CT Patient Population to the Midwest Region; however, descriptive differences in the former two with the latter are apparent. Given this, it should be acknowledged that if the CT Patient Population is not representative of the Midwest Region, then the CT Participant Sample is disadvantaged because the sample can only be drawn from who is available within the healthcare system. Without diverse clinical practices, as represented by the CT Participant Population, the data and conclusions derived from the CT Participant Sample may be compromised, as they may not appropriately reflect the diversity of individuals eligible to participate in CTs.

This paper aimed to assess representativeness of the CT Participant Sample as compared to the CT Patient Population, which represented the target population for representativeness. While the CT Patient Population was used as the comparator group in analysis, results should be interpreted in light of the fact that CT participants would only be sampled from the greater CT Patient Population, which may or may be representative of the Midwest Region population. Given the CT Patient Population includes all patients seen by the PIs of the active CTs during the study time period, individuals in the CT Participant Sample can only be as representative as the CT Patient Population; thus, any non-representativeness could also speak to the fact that CT Patient Population may not be representative of the region in which individuals reside for a number of reasons. Speculating on these reasons are beyond the scope of this paper, but identifying an “ideal” target population is one of the challenges to assessing representativeness, which is why this paper offers readers such a comprehensive approach.

It should be acknowledged that the CT Participant Sample reflect only those individuals who provided informed consent for a CT. Data is not available on individuals who were approached but refused informed consent or on individuals who were eligible but not approached at all. Therefore, it is possible that some of the disparity in representativeness could be explained by differences in individuals who refused participation for various reasons or were otherwise not approached to participate.

## 5 Conclusion

Greater efforts need to be made to enroll participants in CTs that are representative of the healthcare system demographics broadly and the region in which the populations reside. Further, researchers should focus efforts on developing and building upon quantitative frameworks to capture the representativeness of patient samples.

## Data statement

Minimal data needed to interpret, replicate or build upon findings can be made available via request to the corresponding author.

## Funding

This work was supported by an internal grant provided by the Advocate Charitable Foundation under a COVID-19 Relief Funds Mechanism.

## CRediT authorship contribution statement

**Anne Rivelli:** Data curation, Formal analysis, Methodology, Validation, Writing – original draft, Writing – review & editing. **Cheryl Lefaiver:** Conceptualization, Data curation, Investigation, Project administration, Writing – original draft. **Maureen Shields:** Data curation, Investigation, Project administration, Validation. **Osondi Ozoani-Lohrer:** Project administration, Writing – original draft. **Andy Marek:** Data curation, Resources, Validation. **Jana Hirschtick:** Methodology, Writing – original draft. **Veronica Fitzpatrick:** Conceptualization, Data curation, Funding acquisition, Investigation, Methodology, Project administration, Writing – original draft.

## Declaration of competing interest

The authors declare that they have no known competing financial interests or personal relationships that could have appeared to influence the work reported in this paper.

## Data Availability

The authors are unable or have chosen not to specify which data has been used.
